# Clinical outcomes of patients undergoing primary percutaneous coronary intervention for acute myocardial infarction requiring the intensive care unit

**DOI:** 10.1186/s40560-018-0275-y

**Published:** 2018-01-25

**Authors:** Ken Parhar, Victoria Millar, Vasileios Zochios, Emilia Bruton, Catherine Jaworksi, Nick West, Alain Vuylsteke

**Affiliations:** 1 Department of Anesthesia and Intensive Care, Papworth Hospital, Cambridge, England; 2 Department of Interventional Cardiology, Papworth Hospital, Cambridge, England; 3 Department of Critical Care Medicine, University of Calgary, ICU Administration - Ground Floor - McCaig Tower, Foothills Medical Center, 3134 Hospital Drive NW, Calgary, AB T2N 5A1 Canada; 4 Department of Critical Care Medicine, University Hospitals of Birmingham NHS Foundation Trust, Queen Elizabeth Hospital, Birmingham, England

**Keywords:** Acute myocardial infarction, Primary percutaneous coronary intervention, Mechanical ventilation, Intensive care unit

## Abstract

**Background:**

Outcomes for patients with ST-segment elevation myocardial infarction continue to improve, largely due to timely provision of reperfusion by primary percutaneous coronary intervention (PPCI). However, despite prompt and successful PPCI, a small proportion of patients require ventilatory and hemodynamic support in an intensive care unit (ICU). The outcome of these patients remains poorly defined.

**Methods:**

A retrospective review of all consecutive admissions post-PPCI pathway to a single ICU between January 2009 and May 2014 was performed. Patients were analysed based on survival and indication for admission. Preadmission characteristics and ICU course were reviewed. Univariate and multivariable regression analysis was performed to determine predictors of outcome.

**Results:**

During the study period 2902 PPCI were performed and 101 patients were admitted to ICU following PPCI (incidence 3.5%). ICU mortality post-PPCI was 33.7%. Pre-ICU admission factors in a multivariable logistic regression analysis associated with increased mortality included requirement for an intra-aortic balloon pump and a high SOFA score.

**Conclusions:**

ICU admission post PPCI is associated with significant mortality. Mortality was related to high presenting SOFA score and need for IABP. These results provide important prognostic information and an acceptable method for risk-stratifying patients with acute myocardial infarction requiring intensive care.

**Electronic supplementary material:**

The online version of this article (10.1186/s40560-018-0275-y) contains supplementary material, which is available to authorized users.

## Background

Acute myocardial infarction, in particular ST-segment elevation myocardial infarction (STEMI) remains a time-sensitive medical emergency associated with significant morbidity and mortality [1]. In recent years, the widespread recognition of primary percutaneous coronary intervention (PPCI) as an evidence-based treatment strategy that can improve outcomes has led to both an increase in PPCI volume and a reduction in hospital mortality associated with STEMI [2, 3]. A major driver to facilitate this has been the creation and implementation of organised PPCI networks that are able to triage and deliver patients directly to centres able to routinely provide this service both in- and out-of-hours [4, 5]. Patients are subsequently generally cared for in a coronary-care unit (CCU), which has been shown to reduce mortality [6].

The National Infarct Angioplasty Project has demonstrated the benefits of PPCI over thrombolysis for treatment of STEMI patients [7] and has led to the creation of PPCI centres across England. By 2013, some regions demonstrated that more than 95% of patients treated for STEMI received PPCI, compared with only 30% in the third quarter of 2008 [5].

Despite the pervasiveness of PPCI in the management of STEMI and the appropriate use of CCU care, there remains a small proportion of patients that become critically ill and require advanced life support modalities post-PPCI, such as mechanical ventilation or vasoactive therapy that may only be provided within the intensive care unit (ICU). Historically, patients with a complicated myocardial infarction requiring mechanical ventilation have been associated with high rates of morbidity and mortality [8–13].

Patients that may require ICU post-PPCI remain poorly defined. This retrospective single-centre cohort review aims to describe the incidence of admission to ICU, indication for ICU admission, and quantify the morbidity and mortality associated with ICU admission. In addition, factors associated with survival are assessed.

## Methods

### Patient population

We undertook a retrospective review of all consecutive patients admitted to a single tertiary cardiothoracic ICU post-PPCI between January 2009 and May 2014. The unit is the sole provider of intensive care in a subspecialty cardiothoracic hospital serving an English region with a catchment area of approximately three million. All patients requiring PPCI in this region are transferred to this institution.

**Fig. 1:**
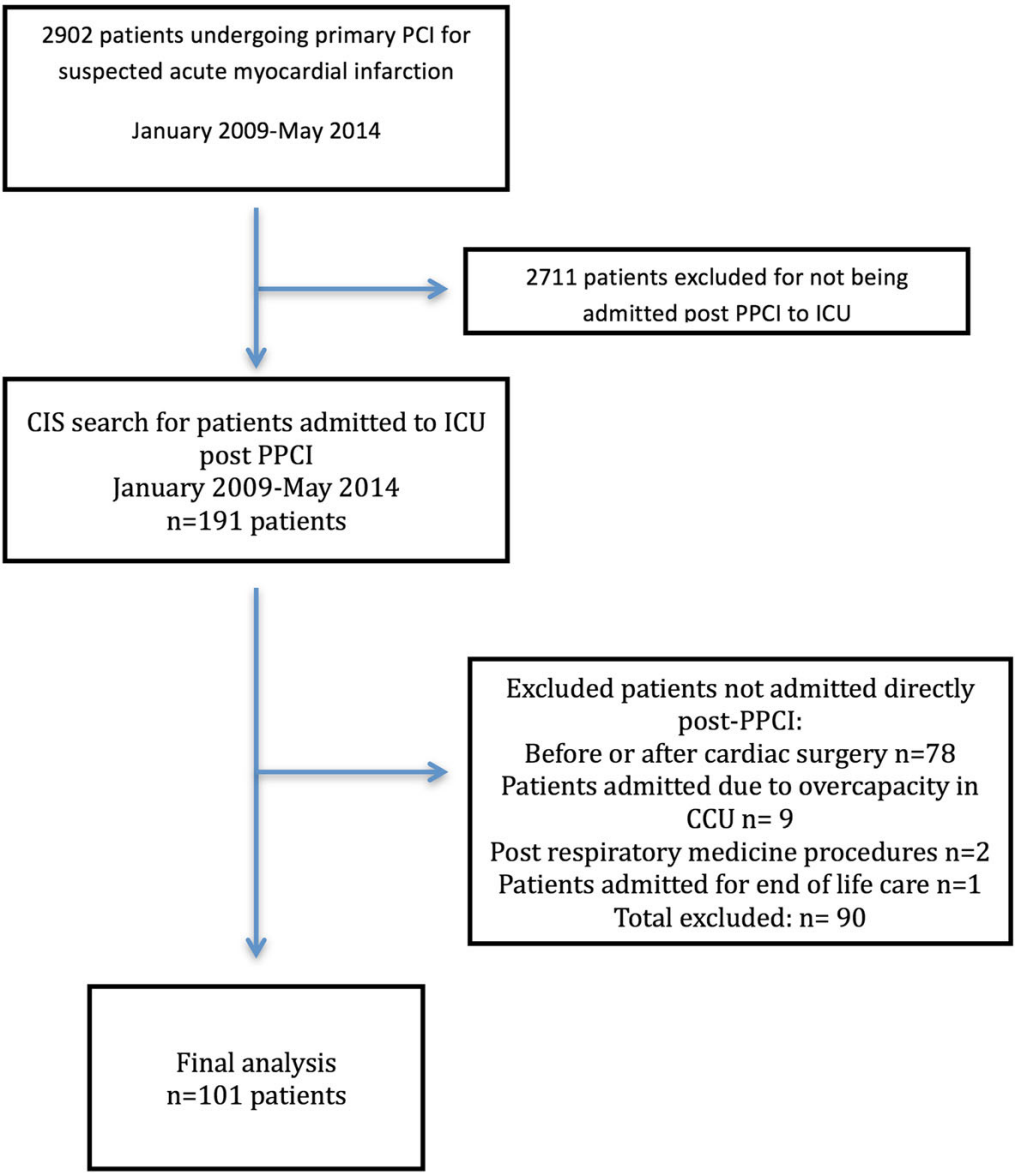
Flowchart outlining patient selection

The search was performed via the electronic Clinical Information System (CIS), which maintains the electronic medical record of all patients admitted to ICU. The initial search yielded 191 patients. Patients were excluded if not admitted directly post-PPCI. Ninety patients were excluded including: patients admitted immediately before or after cardiac surgery or cardiac procedures other than PPCI (*n* = 78), post respiratory medicine procedures (*n* = 2), patients admitted due to lack of beds in CCU (*n* = 9), and patients admitted for end of life care (*n* = 1). A total of 101 patients post-PPCI were appropriate for detailed chart review and analysis (Fig. 1).

### Clinical data

Demographic data (including age, gender, past medical history, and cardiovascular risk factors) were extracted from case-notes and the electronic CIS. Baseline physiological characteristics (vital signs, Glasgow coma scale (GCS), laboratory values) were extracted from the electronic CIS. Details related to PPCI admission, echocardiograms, and cardiac catheterization (downtime, location of infarction, procedures performed, anatomy of coronary disease, complications, door-to-balloon time, pre-PCI interventions) were extracted from a dedicated local database (Philips CVIS, Netherlands), routinely collected for national audits, and patient case-notes where appropriate. ICU interventions, length of stay, and complications were extracted from CIS. Survival data including the ICU and 28 day/hospital outcome was derived from both CIS, case notes and local databases linked to national outcome data.

Vital signs on admission (including heart rate, blood pressure and mean arterial pressure) are reported as mean over the first 24 h of ICU admission. The admission PaO2 to FiO2 (PF) ratio, creatinine, platelets, bilirubin were the worst value measured over the first 24 h. Pulmonary edema was defined as hypoxemia with associated radiographic evidence of interstitial and/or alveolar edema. Cardiogenic shock was defined as a systolic blood pressure < 90 mmHg with clinical evidence of hypoperfusion (cyanosis, mottling, oliguria, cold extremities) or the requirement for an inotrope. New onset renal dysfunction was defined as a 25% rise in serum creatinine or the requirement for renal replacement therapy. The initiation of renal replacement therapy was based on refractory hyperkalemia, refractory acidosis, or volume overload despite medical management. Major hemorrhage was clinical evidence of bleeding with the requirement for four or more units of red blood cells. Infection was a positive culture result, or clinical syndrome consistent with infection such as pneumonia (fever, elevated white cell count, purulent sputum, hypoxemia). Sequential organ function failure assessment (SOFA) score was calculated as previously described [14, 15].

### Groups

Patient outcomes were analysed based on ICU survival. Patient were stratified and analysed based on one of four indications for ICU admission including out-of-hospital cardiac arrest (OHCA), in-hospital cardiac arrest (IHCA), cardiogenic shock, or pulmonary edema. IHCA was defined as a cardiac arrest occurring following arrival to hospital (most commonly during cardiac catheterization), but prior to admission to ICU. Cardiac arrests occurring while in ICU were listed as an ICU complication.

### Statistical analysis

The Shapiro-Wilks test for normality was performed on all continuous variables. Continuous variables with normal distribution were reported as means with standard deviation and analysed by unpaired student’s two-tailed *t* test or one-way analysis of variance (ANOVA) where appropriate. Non-normally-distributed data were reported as median with interquartile range and analysed with the Mann-Whitney *U* test or the Kruskal-Wallis test where appropriate. Categorical variables were analysed with the chi-squared test or Fisher’s exact test where appropriate. A *p* value of < 0.05 was considered statistically significant.

Variables that were statistically significant in the univariate analysis (with a *p* value < 0.10) were considered for inclusion in the multivariable logistic regression model. ICU mortality was defined as the dependant variable. Backward stepwise variable elimination was performed (with a variable exit threshold set at *p* > 0.05). The performance of the final model was assessed using the area under the receiver-operating characteristic (AUROC) curve.

Statistical analysis was performed using Stata Version 13.1 (StataCorp, USA).

### Ethics

Ethical approval was obtained from the Papworth Hospital NHS Foundation Trust research and development board for the completion of this study.

## Results

One-hundred one patients met the inclusion criteria for this retrospective observational study (Fig. 1). During this time, a total of 2902 PPCI were performed, resulting in a post-PPCI incidence of admission to ICU post PPCI of 3.5%.

Of the 101 patients who were admitted to ICU, the majority were male (69%), with a mean age of 65 years (Table 1). Out of hospital cardiac arrest (OHCA) was the most common indication for admission to ICU (36.6%). A significant proportion of patients were admitted for in-hospital cardiac arrest (IHCA; 31.7%) and cardiogenic shock (22.8%). The least common indication for admission to ICU post-PPCI was pulmonary edema (8.9%). Overall ICU mortality was 33.7% for the entire cohort.

**Table 1:** Patient demographic factors for patients admitted to ICU post PPCI. Results are expressed as mean (SD) unless otherwise denoted

	All patients	Outcome			Indication for ICU				
		Survivor	Non-survivor	Sign	OHCA	IHCA	Card shock	Pulm edema	Sign
Total no of patients (%)	101 (100)	67 (66.3)	34 (33.7)		37 (36.6)	32 (31.7)	23 (22.8)	9 (8.9)	
Gender Male, no (%)	70 (69.3)	48 (47.5)	22 (21.8)	0.500	29 (28.7)	20 (19.8)	15 (14.9)	6 (5.9)	0.505
Age, years	65.3 (12.8)	63.8 (11.5)	68.3 (14.8)	0.047	60.2 (12.8)	66.8 (12.6)	71.2 (10.7)	66.0 (11.8)	0.009
Cardiovascular risk factors									
Smoking, no (%)	28 (27.7)	19 (18.8)	9 (8.91)	1.000	11 (10.9)	11 (10.9)	3 (3.0)	3 (3.0)	0.335
Diabetes mellitus, no (%)	21 (20.8)	10 (9.9)	11(10.9)	0.067	4 (4.0)	7 (6.9)	8 (7.9)	2 (2.0)	0.171
Dyslipidaemia, no (%)	30 (29.7)	22 (21.8)	8 (7.9)	0.367	8 (7.9)	10 (9.9)	9 (8.9)	3 (3.0)	0.526
Hypertension, no (%)	58 (57.4)	40 (39.6)	18 (17.8)	0.531	18 (17.8)	17 (16.8)	16 (15.8)	7 (6.9)	0.229
Past medical history									
Previous MI, no (%)	20 (19.8)	15 (14.9)	5 (5.0)	0.436	3 (3.0)	4 (4.0)	8 (7.9)	5 (5.0)	0.002
Previous CAD, no (%)	29 (28.7)	21 (20.8)	8 (7.9)	0.490	4 (4.0)	9 (8.9)	10 (9.9)	6 (5.9)	0.002
Previous CHF, no (%)	3 (3.0)	2 (2.0)	1 (1.0)	1.000	0 (0.0)	1 (1.0)	1 (1.0)	1 (1.0)	0.340
Renal failure, no (%)	12 (11.9)	8 (7.9)	4 (4.0)	1.000	3 (3.0)	2 (2.0)	5 (5.0)	2 (2.0)	0.210
COPD no, no (%)	11 (10.9)	9 (8.9)	2 (2.0)	0.326	3 (3.0)	6 (5.9)	0 (0.0)	2 (2.0)	0.096
Baseline characteristics on admission									
HR (bpm)	79.7 (15.6)	78.5 (16.0)	82.0 (14.5)	0.297	70.6 (15)	80.4 (12.3)	87.8 (13.4)	93.8 (11.7)	< 0.001
Systolic BP (mmHg)	106.8 (19.5)	111.8 (17.9)	96.3 (18.7)	< 0.001	106.9 (16.4)	103.16 (20.7)	108.9 (21.2)	114.0 (22.2)	0.462
MAP (mmHg)	73.0 (13.0)	77.7 (10.3)	63.7 (12.9)	< 0.001	72.5 (12.4)	73.7 (15.3)	71.4 (9.5)	77.0 (15.0)	0.720
PaO2/FiO2 ratio, med (IQR)	143 (98–233)	154 (98–271)	105 (83–173)	0.036	157.9 (105–241)	165 (83–286)	105 (83–143)	128 (75–278)	0.138
GCS, med (IQR)	3 (3–15)	11 (3–15)	3 (3–3)	< 0.001	3 (3–4)	3 (3–14.5)	14 (3–15)	3 (3–15)	0.071
Serum creatinine (μmol/L), med (IQR)	116 (87–157)	102 (84–129)	156 (115–203)	< 0.001	101 (71–126)	116 (95–155)	140 (112–191)	135 (117–144)	0.012
SOFA score	8.4 (3.3)	7.4 (2.9)	10.4 (3.1)	< 0.001	8.6 (2.6)	8.5 (3.5)	8.3 (3.4)	8.0 (5.2)	0.953
Indication for ICU admission									
OHCA, no (%)	37 (36.6)	26 (25.7)	11 (10.9)	0.663					
Downtime before ROSC (min), (IQR)	20 (15–30)	15 (10–20)	35 (30–40)	< 0.001					
IHCA, no (%)	32 (31.7)	19 (18.8)	13 (12.7)	0.368					
Downtime before ROSC (min), (IQR)	10 (5–20)	9 (5–14)	15 (5–42)	0.214					
Cardiogenic shock, no (%)	23 (22.8)	14 (13.9)	9 (8.9)	0.617					
Acute pulmonary oedema, no (%)	9 (8.9)	8 (7.9)	1 (1.0)	0.266					

**Fig. 2:**
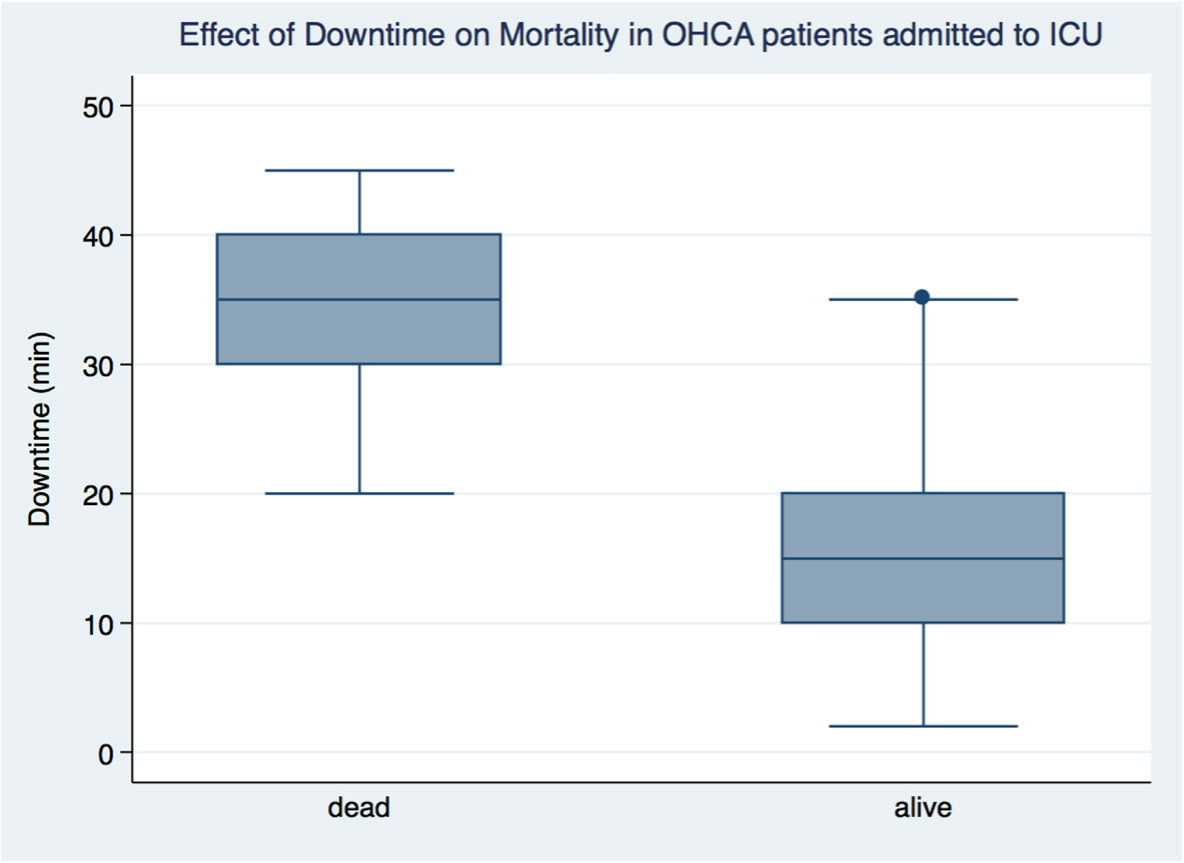
Box and whisker plots of the effect of downtime on return of spontaneous circulation in OHCA patients

Univariate factors that demonstrated a statistically significant difference between survivors and non-survivors included age, low blood pressure on admission (both systolic and mean arterial pressure), low PF ratio, low GCS, high creatinine, and high SOFA scores. In the subgroup of patients suffering from an OHCA, downtime before return of spontaneous circulation (ROSC) was statistically different between survivors and non-survivors. Survivors of ICU post PPCI were associated with a shorter downtime in comparison to non-survivors (Fig. 2). Patients who suffered a witnessed IHCA did not demonstrate a difference in time to ROSC between survivors and non-survivors. When patients were stratified based on their indication for admission (OHCA, IHCA, shock, or pulmonary edema) to ICU post PPCI, there was no difference in mortality amongst the four groups.

STEMI was the most common type of presenting acute coronary syndrome (91%) (Table 2). Other patients who underwent PPCI had either indeterminate ACS (due to a left bundle branch block) or a high suspicion of an evolving transmural infarct. The majority were in the anterior territory (61%) and uncommonly involved the right ventricle (5.0%). Left ventricular (LV) systolic function was depressed in the majority of patients with over 50% of patients having either moderate or severe LV dysfunction as determined by echocardiography during admission. Only one patient received thrombolytics prior to PPCI. Angiogram was successfully performed in the majority of patients (98.0%) with the exception of two patients in whom it was attempted but aborted due to cardiac arrest. There was a high rate of PCI performed (90.1%). Factors that were statistically associated with reduced survival included severe LV dysfunction, right ventricle (RV) involvement, and the need for intra-aortic balloon pump (IABP) insertion in the cath lab. The indications for IABP insertion in the cath lab included cardiogenic shock, bridge for high risk PCI, and ongoing chest pain. IABP were all inserted prior to admission to ICU. The cardiologic factors did not influence the indication for admission to ICU (Additional file 1: Table S1).

**Table 2:** Cardiac characteristics of patients admitted to ICU post PPCI. Results are expressed as mean (SD) unless otherwise denoted

	All Patients	Outcome		
		Survivor	Non-survivor	Sign
Total number of patients (%)	101 (100)	67 (66.3)	34 (33.7)	
STEMI, no (%)	91 (90.1)	58 (57.4)	33 (32.7)	0.158
MI territory				
Anterior, no (%)	61 (61.0)	42 (42.0)	19 (19.0)	0.667
Inferior, no (%)	38 (38.0)	26 (26.0)	12 (12.0)	1.000
Lateral, no (%)	32 (32.0)	22 (22.0)	10 (10.0)	1.000
RV involvement, no (%)	5 (5.0)	1 (1.0)	4 (4.0)	0.040
Peak troponin, ng/L med (IQR)	38.9 (13.7–40.0)	26.8 (10.9–40.0)	40.0 (19.1–626.0)	0.146
LV systolic function				
Normal, no (%)	10 (11.6)	8 (9.3)	2 (2.3)	0.488
Mild dysfunction, no (%)	26 (30.2)	21 (24.4)	5 (5.8)	0.093
Moderate dysfunction, no (%)	22 (25.6)	17 (19.8)	5 (5.8)	0.309
Severe dysfunction, no (%)	28 (32.6)	14 (16.3)	14 (16.3)	0.037
Thrombolysis pre-PPCI, no (%)	1 (1.0)	1 (1.0)	0 (0.0)	1.000
Angiogram, (successful completion) no (%)	99 (98.0)	67 (66.3)	32 (31.7)	0.111
PCI performed (successful completion), no (%)	91 (90.1)	62 (61.4)	29 (28.7)	0.298
IABP in cath lab, no (%)	50 (49.5)	28 (27.7)	22 (21.8)	0.036
Number of diseased vessels, med (IQR)	2 (1–3)	2 (1–3)	2 (2–3)	0.514
Left main stem disease, no (%)	14 (14.1)	8 (8.1)	6 (6.1)	0.371
TIMI flow, med (IQR)	3 (2–3)	3 (2–3)	3 (2–3)	0.862
Symptom onset to device time (min, med (IQR)	210 (155–332)	219 (159–328)	200 (150–350)	0.665

The median duration of stay in the ICU was 3 days (Table 3). Most patients required invasive mechanical ventilation (IMV) (86.1%) with median duration of IMV being 2 days. The majority of the mortality occurred within the ICU (34 of 37 patients). Significant complications were common with patients suffering major bleeding (9.9%), infections (31.7%), acute kidney injury (33.7%), or in ICU cardiac arrest (6.9%). Factors that statistically associated with reduced survival included the lack of use of non-invasive ventilation (NIV), inotropes and vasopressor use, transfusion of blood products including red blood cells (RBCs) and nonRBCs, as well as need for extracorporeal membrane oxygenation (ECMO) or renal replacement therapy (RRT). ECMO was used exclusively in patients who suffered IHCA at any point during the ICU admission. Therapeutic hypothermia was used in patients who suffered either OHCA or IHCA in patients with an initial rhythm of ventricular tachycardia or ventricular fibrillation, but was not associated with a statistically significant increase in survival. There were higher than expected rates of bleeding and transfusions (RBC) in the IHCA group.

**Table 3:** Intensive care characteristics and complications of patients admitted to ICU post PPCI. Results are expressed as mean (SD) unless otherwise denoted

	All patients	Outcome			Indication for ICU				
		Survivor	Non-survivor	Sign	OHCA	IHCA	Card shock	Pulm edema	Sign
Total number of patients (%)	101 (100)	67 (66.3)	34 (33.7)		37 (36.6)	32 (31.7)	23 (22.8)	9 (8.9)	
ICU interventions									
Invasive mechanical ventilation, no (%)	87 (86.1)	56 (55.5)	31 (30.7)	0.373	37 (36.6)	31 (30.7)	12 (11.9)	7 (6.9)	< 0.001
Duration of IMV, median days (IQR)	2 (1–3)	2 (1–2)	2 (1–6)	0.314	2 (2–4)	1 (1–2)	2 (1–5)	1 (1–1)	0.011
Non-invasive ventilation, no (%)	25 (24.8)	21 (20.8)	4 (4.0)	0.049	7 (6.9)	5 (5.0)	8 (7.9)	5 (5.0)	0.047
Duration of NIV, median days (IQR)	1 (1–2)	1 (1–2)	2.5 (1.5–3)	0.075	1 (1–1)	2 (1–2)	2 (1.5–3)	1 (1–1)	0.032
Inotropes, median number (IQR)	1 (0–1)	0 (0–1)	1 (0–2)	0.005	1 (0–1)	1 (0–2)	0 (0–1)	0 (0–1)	0.617
Vasopressors, median number (IQR)	0 (0–1)	0 (0–1)	1 (0–1)	0.001	0 (0–1)	0 (0–1)	0 (0–1)	0 (0–1)	0.971
ECMO, no (%)	7 (6.9)	1 (1.0)	6 (5.9)	0.006	0 (0.0)	7 (6.9)	0 (0.0)	0 (0.0)	0.001
Therapeutic hypothermia, no (%)	48 (47.5)	33 (32.7)	15 (14.9)	0.677	32 (31.7)	14 (13.9)	0 (0.0)	2 (2.0)	< 0.001
IABP, no (%)	59 (58.4)	35 (34.7)	24 (23.8)	0.090	15 (14.9)	23 (22.8)	18 (17.8)	3 (3.0)	0.004
Renal repl therapy, no (%)	27 (26.7)	10 (9.9)	17 (16.8)	< 0.001	6 (5.9)	11 (10.9)	8 (7.9)	2 (2.0)	0.273
In-hospital complications									
Major bleeding, no (%)	10 (9.9)	6 (5.9)	4 (4.0)	0.082	0 (0.0)	7 (6.9)	2 (2.0)	1 (1.0)	0.026
Infections, no (%)	32 (31.7)	21 (20.8)	11 (10.9)	1.000	13 (12.9)	9 (8.9)	7 (6.9)	3 (3.0)	0.936
Renal dysfunction (new onset), no (%)	34 (33.7)	18 (17.8)	16 (15.8)	0.048	12 (11.9)	11 (10.9)	9 (8.9)	2 (2.0)	0.833
In ICU Cardiopulmonary arrest, no (%)	7 (6.9)	0 (0.0)	7 (6.9)	< 0.001	3 (3.0)	4 (4.0)	0 (0.0)	0 (0.0)	0.261
Outcomes									
Duration of ICU stay, median days (IQR)	3 (1–5)	3 (1–4)	2 (0.5–7)	0.389	3 (2–7)	2 (1–5)	3 (0.5–5)	1 (0.5–2)	0.095
ICU mortality, no (%)	34 (33.7)				11 (10.9)	13 (12.9)	9 (8.9)	1 (1.0)	0.346
Hospital / 28-day mortality, no (%)	37 (36.6)				12 (11.9)	14 (13.9)	10 (9.9)	1 (1.0)	0.265
Cause of death (of 37 patients)									
Treatment withdrawn, no (%)	25 (67.6)				7 (18.9)	9 (24.3)	8 (21.6)	1 (2.7)	0.632
Cardiac Arrest, no (%)	7 (18.9)				3 (8.1)	2 (5.4)	2 (5.4)	0 (0.0)	0.867
Other	5 (13.5)				2 (5.4)	3 (8.1)	0 (0.0)	0 (0.0)	0.463

Twenty-eight-day mortality was similar to ICU mortality (Table 3, 36.6 vs 33.7%). The cause of death in most patients was withdrawal of care (67.6%). Post ICU admission cardiac arrest occurred in seven patients (18.9%), none of whom survived.

**Fig. 3:**
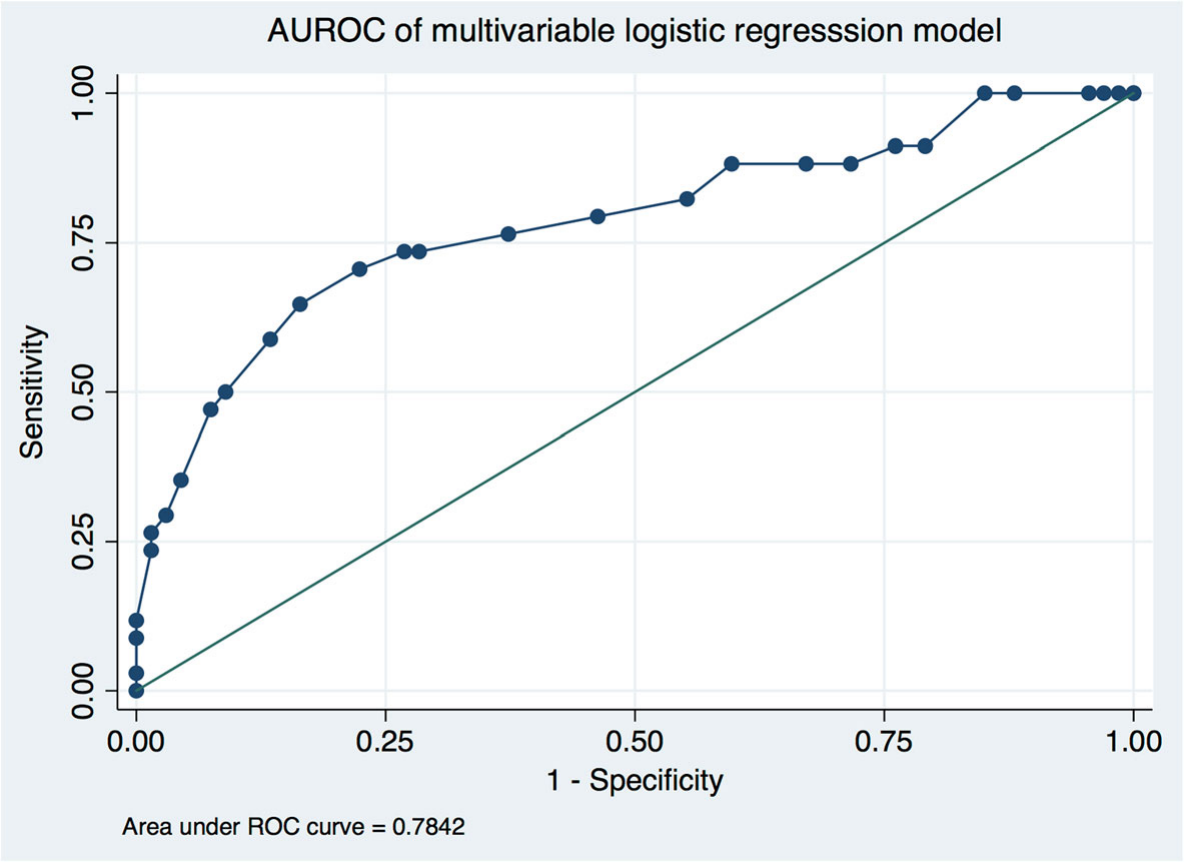
Area under the receiver operating curver (AUROC) of multivariable logistic regression model using IABP and SOFA

Pre-ICU admission factors that demonstrated a statistically significant difference between survivors and non-survivors in univariate analysis were selected for inclusion in multivariable regression analysis. Factors that were independently associated with ICU mortality included high SOFA score and pre-ICU insertion of an IABP (Table 4). Notable factors that were not independently associate with mortality included age, presence of RV dysfunction, and presence of severe LV dysfunction. The odds ratio (OR) for increased mortality for each point increase in SOFA was 1.43 (95% CI 1.2–1.7). The OR for increased mortality when an IABP was inserted pre-ICU admission (during cardiac catheterization) was 3.38 (95% CI 1.27–9.03). The sensitivity and specificity of this model was 50 and 91% respectively with a positive predictive value (PPV) of 73.9% and a negative predictive value (NPV) of 78.2%. This model correctly classified 77.2% of patients in this series and had an AUROC curve of 0.7842 (Fig. 3). When using a model with only SOFA and without IABP, the AUROC was slightly lower (0.75 CI 0.65–0.85) in comparison to the model with SOFA and IABP (0.78 CI 0.68–0.89).

**Table 4:** Multivariable logistic regression analysis of factors associated with ICU mortality

	Odds ratio	Standard error	*z*	*P* value	95% CI
SOFA	1.43	0.127	4.01	0.000	1.200–1.700
IABP	3.38	1.695	2.43	0.015	1.266–9.030

## Discussion

In this retrospective observational study, we present a series of consecutive patients post-PPCI pathway that are critically ill and require admission to the ICU for advanced therapies that may only be provided in ICU such as invasive mechanical ventilation or vasoactive support. There is a significant mortality amongst these patients (33.6%), which is significantly higher than the general PPCI population. Indication for admission (cardiac arrest, pulmonary edema, cardiogenic shock) does not statistically influence mortality and all groups were similar despite their indication. Those patients presenting with higher SOFA scores (reflecting a higher degree of multiple-organ dysfunction), or requiring an IABP during cardiac catheterization were independently associated with higher mortality.

Despite an era of appropriate anti-ischemic therapy post-STEMI and provision of organised and timely reperfusion via PPCI, there remain a proportion of patients who become critically ill and require admission to ICU for invasive monitoring, mechanical ventilation or vasoactive therapy. Patient mortality in this group remains high despite improving outcome for all patients with STEMI presenting for PPCI [3].

In contrast to previous studies of patients requiring mechanical ventilation or suffering from cardiogenic shock following complicated myocardial infarction, our study reviewed consecutive patients admitted to ICU exclusively via the PPCI pathway [8–13, 16, 17]. This included both patients who required mechanical ventilation and those who did not.

A higher SOFA score was associated with increased mortality (Table 4). This suggests that degree of organ dysfunction in patients with complicated myocardial infarction, as with many other critical illnesses, is a major determinant of survival. Surprisingly, neither the requirement for mechanical ventilation nor the indication for admission were independently associated with mortality. To date, no study has described this relationship exclusively in the post-PPCI patient population. A previous study looking at traditional risks scores used in the myocardial infarction population such as the Global Registry of Acute Coronary Events (GRACE) risk score or the Thrombolysis in Myocardial Infarction (TIMI) risk score in comparison with SOFA demonstrated that SOFA provided reasonable discrimination of prognosis [18]. This study was limited, as it did not focus on the post-PPCI population or those patients specifically who were admitted to ICU, which are most likely to be critically ill and potentially benefit from prognostication. Our study is novel as we demonstrate that the SOFA score does predict mortality in this high-risk group of patients admitted to the ICU who require mechanical ventilation and vasopressors. Previous studies looking specifically at patients admitted to ICU with cardiogenic shock demonstrated that there was an association between scores such as Acute Physiology and Chronic Health II/III (APACHE II/III), Simplified Acute Physiology Score II (SAPSII), SOFA and survival outcome [16, 17]. The specific organ systems within the SOFA score that were responsible for the higher scores included increased renal dysfunction, lower admission GCS, as well as worse hypoxemia (lower PaO2/FiO2 ratio (Table 1). The benefit of using SOFA scores and the presence or absence of IABP to prognosticate patients is that it can be easily calculated upon admission to ICU with information routinely available. This is the drawback of scores such as APACHE II/III and SAPSII as they are more complex and time consuming to calculate when compared to SOFA [19]. SOFA was used as a prognostic score due to its simplicity and ability to be calculated with very routine and objective patient data. Retrospective data collection made it difficult to use alternate scores such as APACHE II due to the high number of variables required in these scores including patient historical factors and the risk of missing data [19]. For example, any missing data precluded patients from being included in APACHE II score calculation as per the original description of APACHE II [20]. In addition, it has been demonstrated that there are significant differences in the ability of APACHE II to be calculated accurately when comparing prospective and retrospective collection of data [21]. This further highlights the strength of the SOFA score as it is quick and easy to calculate using commonly available objective clinical data.

We demonstrated that use of IABP was independently associated with mortality which is in keeping with previously reported observational data [22, 23]. The cohort of the study patients who required mechanical circulatory support with IABP and ICU admission was representative of the higher risk patient population and therefore IABP support may have been given to the sicker patients which would induce bias towards poor outcomes in that group. A meta-analysis of cohort studies in the context of STEMI leading to cardiogenic shock supported the use of IABP adjunctive to fibrinolysis [24]. It remains unknown whether early IABP placement can improve clinically important outcomes in patients with STEMI requiring ICU admission.

In the subgroup of patients with an OHCA, a longer down time before ROSC was associated with higher mortality; however, in the multivariable analysis, this was not an independent predictor for increased mortality. The association between prompt ROSC and outcome has been well described previously [25]. Similarly age was a univariate factor associated with increased mortality however was not an independent predictor in the multivariable analysis. It may be that increased age and longer downtimes before ROSC are all reflective of increased likelihood of organ dysfunction and a higher SOFA score, thus not independently associated with mortality.

In the IHCA group, there were five patients who were supported with ECMO under cardiac arrest conditions (E-CPR). The IHCA group had a high rate of major bleeding most likely associated with the use of E-CPR, as this association is a well described in other ECMO populations [26]. Survival in this group was low which is consistent with previously published reviews on the use of E-CPR in this age demographic [27].

This study had several limitations. It was performed at a single tertiary PPCI referral centre and is retrospective in nature and thus data collection was based on review of the CIS and paper charts. The multivariable logistic regression model was not externally validated in an alternate population or in patients not admitted to ICU. Furthermore, there may be selection bias for patients requiring mechanical circulatory support with IABP due to differences in individual clinical practice patterns.

This study provides a rationale for a future prospective observational study and validation of the multivariable model to determine if this may help triage and prognosticate patients who are not likely to survive post complicated acute myocardial infarction. Potential uses for this type of model include being able to provide prognostic information for care providers and patient family members. It may also help identify patients in whom aggressive care may be deemed unlikely to succeed. Alternatively, if these patients are identified correctly a priori, it may allow a targeted intervention to improve outcomes in this cohort of patients who continue to have an extremely poor outcome.

## Conclusions

Despite only requiring admission 3.5% of the time to ICU (101 of 2902 patients), those patients suffering an MI that do require ICU post PPCI are very critically ill and have a mortality of 33.7%. The most effective way to prognosticate survival in this cohort of patients is by using the SOFA score, in addition to the requirement for an intra-aortic balloon pump.

## Additional file

Additional file 1: Table S1. Cardiac characteristics of patients admitted to ICU post PPCI by indication. Results are expressed as mean (SD) unless otherwise denoted. (DOCX 17 kb)

## Abbreviations

ANOVA: Analysis of variance
APACHE: Acute physiology and chronic health
AUROC: Area under the receiver operating curve
CAD: Coronary artery disease
CCU: Coronary care unit
CIS: Clinical information system
ECMO: Extracorporeal membrane oxygenation
E-CPR: Ecmo during cardio-pulmonary resuscitation
GCS: Glasgow coma scale
IABP: Intra-aortic balloon pump
ICU: Intensive care unit
IHCA: In-hospital cardiac arrest
IMV: Invasive mechanical ventilation
LV: Left ventricle
NIV: Non-invasive mechanical ventilation
NPV: Negative predictive value
OHCA: Out of hospital cardiac arrest
OR: Odds ratio
PF: PaO2:FiO2
PPCI: Primary percutaneous coronary intervention
PPV: Positive predictive value
RBC: Red blood cells
ROSC: Return of spontaneous circulation
RRT: Renal replacement therapy
RV: Right ventricle
SAPSII: Simplified acute physiology score II
SOFA: Sequential organ failure assessment
STEMI: ST-segment elevation myocardial infarction
