# Correction to: Clinical outcomes of patients undergoing primary percutaneous coronary intervention for acute myocardial infarction requiring the intensive care unit

**DOI:** 10.1186/s40560-018-0301-0

**Published:** 2018-05-31

**Authors:** Ken Parhar, Victoria Millar, Vasileios Zochios, Emilia Bruton, Catherine Jaworski, Nick West, Alain Vuylsteke

**Affiliations:** 1 Department of Anesthesia and Intensive Care, Papworth Hospital, Cambridge, England; 2 Department of Interventional Cardiology, Papworth Hospital, Cambridge, England; 3 Department of Critical Care Medicine, University of Calgary, ICU Administration - Ground Floor - McCaig Tower, Foothills Medical Center, 3134 Hospital Drive NW, Calgary, AB T2N 5A1 Canada; 4 Department of Critical Care Medicine, University Hospitals of Birmingham NHS Foundation Trust, Queen Elizabeth Hospital, Birmingham, England

## Correction

In the original publication of this article [1], one author’s name was misspelling. Catherine Jaworksi should be Catherine Jaworski.
