# Supplementary material for: Clinical outcomes of patients undergoing primary percutaneous coronary intervention for acute myocardial infarction requiring the intensive care unit
Source: J Intensive Care. 2018 Jan 25;6:5. doi: 10.1186/s40560-018-0275-y (PMC5784703; doi:10.1186/s40560-018-0275-y)
Supplement: Additional file 1: Table S1. — Cardiac characteristics of patients admitted to ICU post PPCI by indication. Results are expressed as mean (SD) unless otherwise denoted. (DOCX 17 kb) [file 40560_2018_275_MOESM1_ESM.docx]

|  | All Patients | Indication for ICU | | | | |
| --- | --- | --- | --- | --- | --- | --- |
|  |  | OHCA | IHCA | Card Shock | Pulm Edema | Sign |
|  |  |  |  |  |  |  |
| Total number of patients (%) | 101 (100) | 37 (36.6) | 32 (31.7) | 23 (22.8) | 9 (8.9) |  |
| STEMI, no (%) | 91 (90.1) | 35 (34.7) | 31 (30.7) | 19 (18.8) | 6 (5.9) | 0.024 |
| MI territory |  |  |  |  |  |  |
| Anterior, no (%) | 61 (61.0) | 25 (25.0) | 19 (19.0) | 13 (13.0) | 4 (4.0) | 0.727 |
| Inferior, no (%) | 38 (38.0) | 11 (11.0) | 13 (13.0) | 11 (11.0) | 3 (3.0) | 0.550 |
| Lateral, no (%) | 32 (32.0) | 10 (10.0) | 13 (13.0) | 7 (7.0) | 2 (2.0) | 0.632 |
| RV involvement, no (%) | 5 (5.0) | 2 (2.0) | 1 (1.0) | 2 (2.0) | 0 (0.0) | 0.722 |
| Peak troponin, median (IQR) | 38.9 (13.7-40.0) | 38.9 (14.9-296.5) | 40.0 (8.6-40.0) | 31.5 (18.2-40.0) | 16.5 (0.6-40.0) | 0.290 |
| LV systolic function |  |  |  |  |  |  |
| Normal, no (%) | 10 (11.6) | 4 (4.0) | 3 (3.0) | 3 (3.0) | 0 (0.0) | 0.732 |
| Mild dysfunction, no (%) | 26 (30.2) | 10 (9.9) | 12 (11.9) | 3 (3.0) | 1 (1.0) | 0.151 |
| Moderate dysfunction, no (%) | 22 (25.6) | 8 (7.9) | 2 (2.0) | 9 (8.9) | 3 (3.0) | 0.026 |
| Severe dysfunction, no (%) | 28 (32.6) | 8 (7.9) | 11 (10.9) | 5 (5.0) | 4 (4.0) | 0.382 |
| Thrombolysis pre-PPCI, no (%) | 1 (1.0) | 0 (0.0) | 1 (1.0) | 0 (0.0) | 0 (0.0) | 0.529 |
| Angiogram, (success compl) no (%) | 99 (98.0) | 37 (36.6) | 32 (31.7) | 22 (21.8) | 8 (7.9) | 0.115 |
| PCI (success compl), no (%) | 91 (90.1) | 35 (34.7) | 32 (31.7) | 17 (16.8) | 7 (6.9) | 0.005 |
| IABP in cath lab, no (%) | 50 (49.5) | 15 (14.9) | 19 (18.8) | 13 (12.9) | 3 (3.0) | 0.280 |
| No of diseased vessels, median (IQR) | 2 (1-3) | 2 (2-3) | 2 (1-3) | 2 (1-3) | 2 (2-3) | 0.807 |
| Left main stem disease, no (%) | 14 (14.1) | 2 (2.0) | 7 (7.1) | 3 (3.0) | 2 (2.0) | 0.196 |
| TIMI flow, median (IQR) | 3 (2-3) | 3 (3-3) | 3 (2-3) | 3 (2-3) | 3 (2-3) | 0.748 |
| Symp to device time (min) med (IQR) | 210 (155-332) | 190 (138-282) | 210 (150-380) | 240 (180-335) | 346 (163-469) | 0.377 |

**Additional file 1: TABLE S1** - Cardiac characteristics of patients admitted to ICU post PPCI. Results are expressed as mean (SD) unless otherwise denoted.
